# Docking domain-mediated subunit interactions in natural product megasynth(et)ases

**DOI:** 10.1093/jimb/kuab018

**Published:** 2021-02-26

**Authors:** Helen G Smith, Matthew J Beech, Józef R Lewandowski, Gregory L Challis, Matthew Jenner

**Affiliations:** Warwick Medical School, University of Warwick, Coventry CV4 7AL, UK; Department of Chemistry, University of Warwick, Coventry CV4 7AL, UK; Department of Chemistry, University of Warwick, Coventry CV4 7AL, UK; Department of Chemistry, University of Warwick, Coventry CV4 7AL, UK; Department of Chemistry, University of Warwick, Coventry CV4 7AL, UK; Warwick Integrative Synthetic Biology Centre, University of Warwick, Coventry CV4 7AL, UK; Department of Biochemistry and Molecular Biology, Biomedicine Discovery Institute, Monash University, Clayton, VIC 3800, Australia; ARC Centre of Excellence for Innovations in Peptide and Protein Science, Monash University, Clayton, VIC 3800, Australia; Department of Chemistry, University of Warwick, Coventry CV4 7AL, UK; Warwick Integrative Synthetic Biology Centre, University of Warwick, Coventry CV4 7AL, UK

**Keywords:** Polyketide synthase, Non-ribosomal peptide synthetase, Biosynthesis

## Abstract

Polyketide synthase (PKS) and non-ribosomal peptide synthetase (NRPS) multienzymes produce numerous high value metabolites. The protein subunits which constitute these megasynth(et)ases must undergo ordered self-assembly to ensure correct organisation of catalytic domains for the biosynthesis of a given natural product. Short amino acid regions at the N- and C-termini of each subunit, termed docking domains (DDs), often occur in complementary pairs, which interact to facilitate substrate transfer and maintain pathway fidelity. This review details all structurally characterised examples of NRPS and PKS DDs to date and summarises efforts to utilise DDs for the engineering of biosynthetic pathways.

## Introduction

Modular polyketide synthases (PKSs) and non-ribosomal peptide synthetases (NRPSs) represent two classes of extraordinary molecular machines, responsible for the biosynthetic assembly of polyketide and non-ribosomal peptide natural products, respectively. Over the past 30 years, the chemical products of these ‘megaenzymes’ have been the focus of extensive research due to the potential of these molecules as sources of new pharmaceuticals and agrochemicals (Newman & Cragg, 2020). Frequently equated to molecular assembly lines, modular PKSs and NRPSs typically consist of multiple large protein subunits comprised of discretely-folded catalytic domains organised into modules with overall molecular weights in the MDa range (Weissman & Muller, 2008). Both PKS and NRPS paradigms employ carrier protein domains; an acyl carrier protein (ACP) domain in modular PKSs and a peptidyl carrier protein (PCP) domain in NRPSs, which are post-translationally modified via tethering of a 4′-phosphopantetheine (Ppant) group to a conserved serine residue (Crosby & Crump, 2012). This facilitates covalent attachment of the biosynthetic intermediates via a thioester linkage, whilst also providing the required flexibility to visit active sites of other enzymatic domains within the module (Fischbach & Walsh, 2006; Hertweck, 2009).

**Fig. 1.**
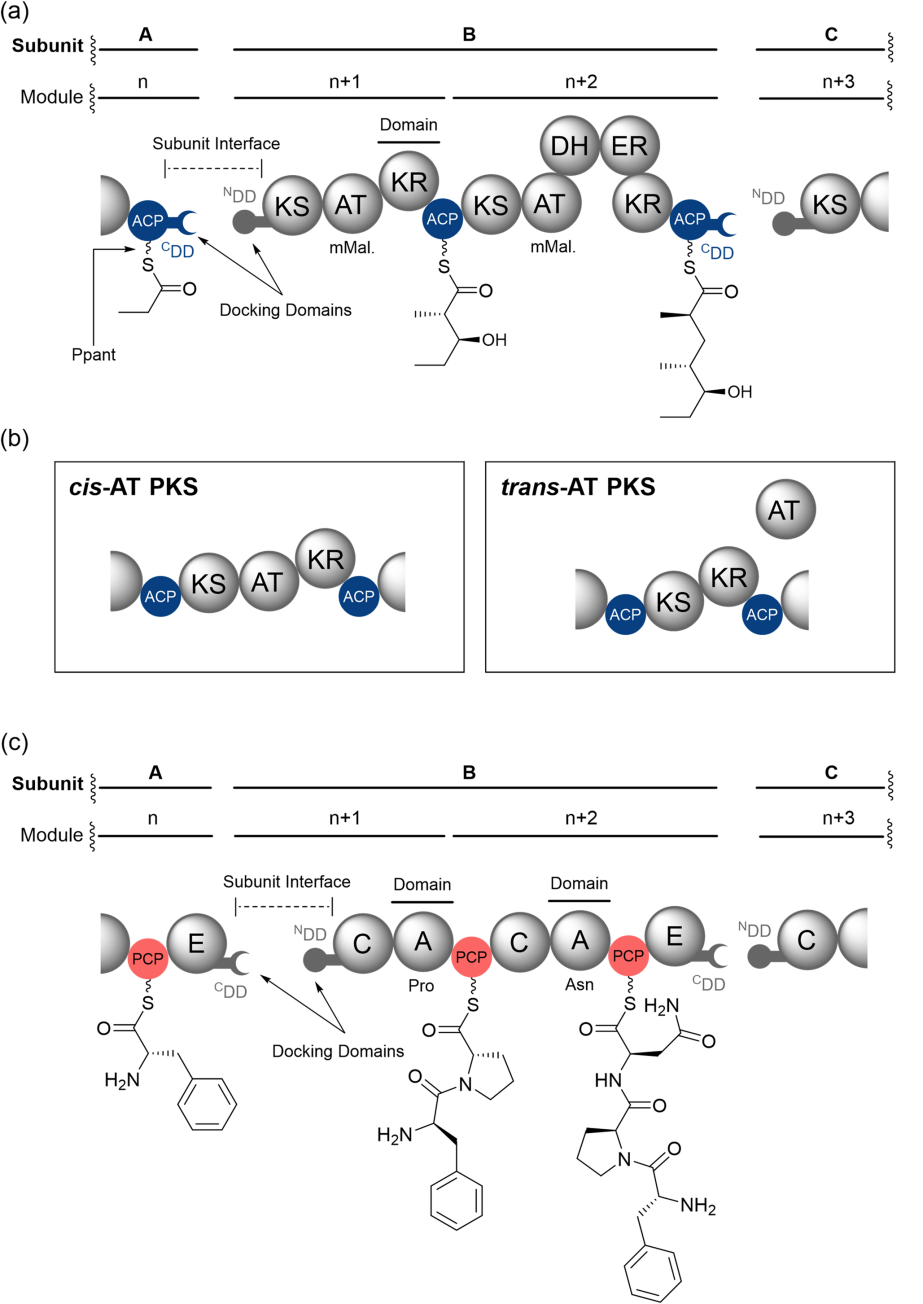
(a) Partial domain organisation of a hypothetical PKS assembly line. Enzymatic domains are represented by spheres and biosynthetic intermediates are shown appended to each ACP domain. Subunit and module labelling conventions are highlighted above the PKS, and features relating to the main text are highlighted. (b) Domain organisation of *cis*-AT (*left*) and *trans*-AT (*right*) PKS modules. The stand-alone AT domain in *trans*-AT PKSs loads extender units onto multiple ACP domains, whereas the AT domain in *cis*-AT PKSs loads the ACP domain within its module. (c) Partial domain organisation of a hypothetical NRPS assembly line. Enzymatic domains are represented by spheres and biosynthetic intermediates are shown appended to each PCP domain. Subunit and module labelling conventions are highlighted above the NRPS, and features relating to the main text are highlighted. Domain abbreviations are as follows: KS, ketosynthase; AT, acyltransferase; DH, dehydratase; ER, enoylreductase; KR, ketoreductase; ACP, acyl carrier protein; C, condensation; A, adenylation; PCP, peptidyl carrier protein; ^C^DD, C-terminal docking domain; ^N^DD, N-terminal docking domain.

Polyketide biosynthesis involves the head-to-tail condensation of acyl and malonyl-derived thioester units, similar to the catalytic cycle of fatty acid biosynthesis (Smith & Tsai, 2007; Staunton & Weissman, 2001). During polyketide biosynthesis the catalytic cycle of a module commences when an acyltransferase (AT) domain loads an (alkyl)malonyl extender unit onto the Ppant thiol of the ACP domain. The ketosynthase (KS) domain then catalyses a decarboxylative Claisen condensation between an incoming extender unit and the upstream acylthioester intermediate, yielding a β-keto-thioester. Optional catalytic domains within the module, such as ketoreductase (KR), dehydratase (DH), enoylreductase (ER) and methyltransferase (MT) domains, allow modification of the α- and β-carbons of the β-keto-thioester (Keatinge-Clay, 2012; Khosla et al., 2007) (Fig. 1a). Modular PKSs can be sub-divided into two phylogenetically distinct classes; the *cis*-AT and *trans*-AT PKSs. In *cis*-AT PKS systems an AT domain is integrated into each module of the PKS. In *trans*-AT PKSs the modules lack AT domains and a stand-alone AT acts *in trans* to functionalise each ACP domain (Helfrich & Piel, 2016; Kosol et al., 2018) (Fig. 1b).

NRPSs employ a similar biosynthetic logic to modular PKSs, where each module harbours the enzymatic domains required for selection, activation and incorporation of amino acid building blocks into the growing peptidyl chain (Reimer et al., 2018; Süssmuth & Mainz, 2017). In this case, an adenylation (A) domain specifically selects and activates an amino acid via adenylation of the carboxyl group, permitting subsequent tethering to the Ppant thiol of the PCP domain (Challis et al., 2000; Stachelhaus et al., 1999). A condensation (C) domain then catalyses peptide bond formation between the PCP-bound aminoacyl thioester and the peptidyl thioester intermediate attached to the upstream PCP domain (Fig. 1c). Common optional domains in NRPS machinery include an epimerisation (E) domain, which catalyses inversion of the stereochemistry at the α-carbon, and an N-methyltransferase (N-MT) domain that methylates the nitrogen of the peptide linkage (Miller & Gulick, 2016).

In both PKSs and NRPSs, the catalytic domains are often split across multiple large protein subunits. These subunits must undergo ordered self-assembly to ensure the directionality of the assembly line is maintained, and to guarantee biosynthetic fidelity. Short regions of amino acids situated at the N- and C-termini of multi-modular subunits have been shown to direct ordered assembly of both PKSs and NRPSs (Broadhurst et al., 2003; Kosol et al., 2018; Richter et al., 2008) (Fig. 1a and c). These regions are known as docking domains (DDs), and tend to occur in complementary pairs that interact in the low µM range, but specifically enough to maintain biosynthetic fidelity (Dodge et al., 2018; Kosol et al., 2018; Miyanaga et al., 2018). Their discovery has presented a unique opportunity to expand the combinatorial potential of PKS and NRPS assembly lines via grafting of DD pairs onto heterologous modules to create novel chemical scaffolds. However, to achieve success in such bioengineering experiments, the nature of the docking interfaces needs to be understood in molecular detail.

Over the last 20 years, there have been several efforts to elucidate the structures and molecular interaction mechanisms involved in modular PKS and NRPS DD interfaces. Structural studies of DDs are often undertaken via NMR spectroscopy or X-ray crystallography and have required production of a covalently fused C-terminal DD (^C^DD)–N-terminal DD (^N^DD) complex, as one or both DDs within the pair may only fold correctly in the presence of its cognate partner (Broadhurst et al., 2003). More recently, newer techniques such as small angle X-ray Scattering (SAXS) (Dorival et al., 2016; Risser et al., 2020) and carbene footprinting mass spectrometry have been employed (Jenner et al., 2018; Kosol et al., 2019), alongside traditional techniques to elucidate both DD structure and molecular interactions acting across the interface.

This review details all characterised examples of NRPS and PKS DDs to date and summarises efforts to utilise these to engineer PKS and NRPS biosynthetic pathways. The potential of DDs to be exploited for assembly line engineering is considerable, and as the mechanisms underpinning DD interactions are further elucidated it is expected that their use in pathway engineering will be expanded to a wider variety of systems.

## PKS DDs

PKS DDs can be categorised by the type of system they occur in; *cis*- or *trans*-AT PKSs. It is worth noting that the subunit junctions of *cis*-AT PKSs are predominantly intermodular, resulting in many examples of ACP/KS domain junctions. *Trans*-AT PKSs, on the other hand, tend to have intramodular subunit junctions giving rise to multiple ‘split module’ architectures (e.g. KS/KR, KS/DH and DH/KR domain junctions) (Helfrich & Piel, 2016). Perhaps because of this, there is more structural diversity in the type of DDs that occur in *trans*-AT PKS systems.

### *Cis-*AT PKS DDs

*Cis-*AT PKS DDs are split into classes according to the type of organism that produces the metabolite; Class 1 DDs have been proposed to be present primarily in Actinobacterial modular PKSs, whereas Class 2 DDs are proposed to be found predominantly in modular PKSs from Cyanobacteria, Myxobacteria and other Gram-negative bacteria (Broadhurst et al., 2003; Buchholz et al., 2009; Whicher et al., 2013). Class 2 *cis*-AT PKS DDs are found both in purely PKS systems and in hybrid PKS–NRPSs, but will be discussed in this section (Whicher et al., 2013).

#### Class 1 cis-AT PKS DDs: Four α-helix bundles

**Fig. 2.**
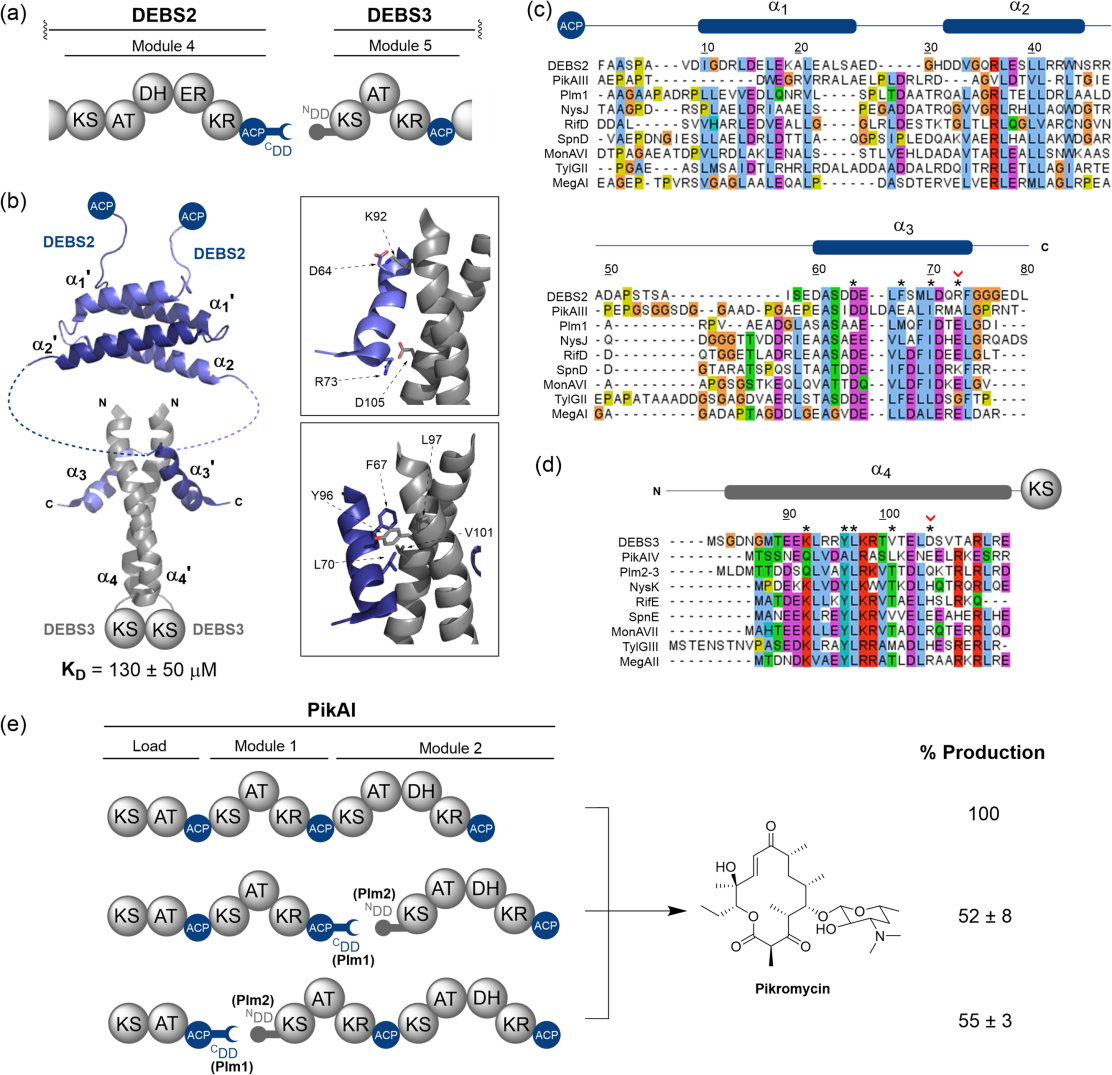
Structural features, sequence alignments and use in biosynthetic engineering of Class 1 PKSs DDs. (a) Domain architecture of the DEBS2–DEBS3 intersubunit junction from the PKS involved in the biosynthesis of erythromycin A. (b) Solution state NMR structure of the 4-α-helix bundle docked complex formed by the covalently tethered DEBS2 C-terminal and DEBS3 N-terminal DDs (PDB accession code: 1PZR). A dimerisation motif is found upstream of the docking interface and comprises four additional helices (PDB accession code: 1PZQ). *Inset (top)*: Key electrostatic interactions between helix α_3_ and helices α_4_ and α_4_′ that confer specificity to the docking interface. *Inset (bottom)*: Hydrophobic interface formed between the α_3_, α_4_ and α_4_′ helices. (c) Sequence alignment of select 4-α-helix bundle C-terminal docking domains (DDs), including the dimerisation motif. (d) Sequence alignment of select 4-α-helix bundle N-terminal DDs. Asterisks (*) denote the positions of the interfacial residues highlighted in (b). Red chevrons (^v^) denote interacting electrostatic residues highlighted in (b) where charge is not conserved across DDs. Above the alignments, a schematic displaying the positions of the secondary structural elements from the solution state NMR structure of the DEBS2–DEBS3 DD complex is provided. Residue numbering provided in (b), (c) and (d) is relative to that from PDB accession code 1PZQ. (e) Artificial splitting of the PikAI subunit from the PKS responsible for pikromycin biosynthesis using four α-helix bundle DDs from the Plm1–Plm2 subunit junction in phoslactomycin biosynthesis. Quantification of pikromycin production was determined by HPLC analysis of culture extracts from a *Streptomyces venezuelae* Δ*pikAI* mutant complemented with the engineered PikAI proteins *in trans*.

Class 1 PKS DDs occur exclusively at ACP/KS subunit junctions, with complementary DD regions at the C-terminus of the ACP domain (^C^DD), and the N-terminus of the KS domain (^N^DD). Three α-helices (α_1_–α_3_) comprise the ^C^DD region; α_1_ and α_2_ form a dimerisation element, and α_3_ is directly involved in contacts with the ^N^DD (Broadhurst et al., 2003). It is worth noting that, unlike NRPS systems, PKSs are dimeric and consequently a total of six α-helices makes up the ^C^DD region. The ^N^DD connected to the downstream KS domain consists of a single α-helix (α_4_), which forms a coiled–coil with an identical ^N^DD in the dimeric KS (Fig. 2a and b). The shorter α_3_ helices from the ^C^DD interact with the ^N^DD by clamping to each side of the coiled–coil to form a four α-helix bundle complex (Broadhurst et al., 2003) (Fig. 2b). Structurally characterised examples of actinobacterial Class 1 PKS DDs include the DEBS2/DEBS3 interface from the erythromycin PKS (Broadhurst et al., 2003) and the PikAIII/PikAIV interface from the pikromycin PKS (Buchholz et al., 2009). Interestingly, a Class 1 DD has also been characterised from the Bamb_5920/Bamb_5919 interface from the enacyloxin IIa hybrid PKS–NRPS, in Gram-negative *Burkholderia* species (Risser et al., 2020) (Fig. 2).

The DEBS2/3 DD pair was the first to be structurally characterised. Covalently tethered ^C^DD(α_1_–α_2_) and ^C^DD(α_3_)–^N^DD constructs were analysed by solution state NMR spectroscopy and both the structure and molecular interactions across the interface were elucidated. Interactions between the ^N^DD α-helices forming the dimeric coiled–coil were found to be predominantly hydrophobic, though some salt bridges were present. Sequence alignments with other Class 1 ^N^DDs showed that the residues involved in salt bridge formation were conserved across systems, indicative of their importance in creation of the coiled–coil interaction interface. Important interactions between the ^C^DD (α_3_) and ^N^DD of this interface were also identified as involving a combination of hydrophobic contacts and salt bridges. Hydrophobic contacts highlighted in Fig. 2b were found to be highly conserved across multiple Class 1 DDs. Examples include F67 on α_3_, Y96 on α_4_ and L97 on α_4_′ (Fig. 2c and d). Further sequence alignments led to the hypothesis that the salt bridges, such as those formed between R73 of α_3_ and D105 of α_4_, confer specificity to the interface. On interaction of non-cognate DDs, repulsive interactions would occur at these positions, this would prevent docking of non-cognate pairs and ensure biosynthetic fidelity is maintained (Broadhurst et al., 2003).

Structural and interaction data for the PikAIII/PikAIV DD pair were obtained via crystallisation of a covalently tethered ^C^DD–^N^DD complex. The structure and key amino acid contacts, both between the ^C^DD and ^N^DD and between the two α-helices of the coiled–coil, were found to be similar to that characterised from the erythromycin system. However, the specificity conferring ionic contact between R73 and D105 in the DEBS2-3 system is not maintained. The PikAIII ^C^DD is shorter than the DEBS2 ^C^DD, at only nine amino acids compared to fifteen. It is therefore understandable that electrostatic interactions across the interface may occur at alternate positions. Sequence alignments of DEBS2 and PikAIII ^C^DDs with other ^C^DDs from the erythromycin and pikromycin biosynthetic pathways found that charge reversal of a single amino acid residue was enough to confer specificity to these interfaces. This highlights the importance of these electrostatic interactions in driving ordered self-assembly of the modular PKS subunits (Buchholz et al., 2009).

A covalently tethered ^C^DD–^N^DD complex also enabled structure elucidation of the Bamb_5920/5919 DD pair from the enacyloxin IIa biosynthetic system. Solution state NMR spectroscopy revealed a complex with a highly similar structure to those previously elucidated. However, the Bamb_5920 ^C^DD lacks the double helix dimerisation motif present in the erythromycin system (Fig. 2b), indicating that the dimerisation motif may not be essential for docking. The Bamb_5920/5919 docking interface was, as with other Class 1 DDs, found to interact via a combination of hydrophobic contacts and salt bridges. However, attempts to align these DD sequences with other characterised examples were problematic and highlighted their divergent nature (Fig. 2c and d). This could perhaps be a consequence of the DD originating from a hybrid PKS–NRPS, leading to evolutionary divergence at the sequence level despite high structural similarity. Nevertheless, as this DD is the first characterised example of a Class 1 DD pair identified in a hybrid PKS–NRPS it offers the potential to facilitate the production of hybrid biosynthetic pathways. As further examples of this class of DD are identified in hybrid systems, more will be understood about their modes of interaction and specificity-conferring mechanisms (Risser et al., 2020).

Class 1 PKS DDs were identified via sequence analysis several years prior to elucidation of the structure of the DEBS2-3 DD pair. Many early experiments were undertaken on the DEBS PKS both at the DEBS1-2 and the DEBS2-3 interaction interface to better understand the role of DDs. These experiments included overproduction of modules lacking their DDs. These remained functional, showing their non-essentiality for modular PKS function *in vitro* (Broadhurst et al., 2003). Replacement of native DD pairs with cognate pairs from other biosynthetic pathways allowed *in vitro* interaction of modules with a K_D_ almost identical to the wild-type system (Broadhurst et al., 2003). Cognate DD pairs were found to enable *in vitro* interaction of modules from different biosynthetic pathways, as demonstrated by creation of a hybrid rifamycin-DEBS PKS (Gokhale et al., 1999). Introduction of a non-cognate partner DD to a modular PKS interface caused a large decrease in product titre, but surprisingly production was not completely abolished (Tsuji et al., 2001). This provided preliminary evidence that more than just interaction of DD pairs is necessary for productive protein–protein interaction and substrate transfer across subunit junctions. At the point the first structure of a DD pair was elucidated, much was already known about both the portability and necessity of these interacting regions.

More recently, *in vivo* pathway engineering was attempted on the pikromycin PKS. A cognate DD pair was found to facilitate interaction between modules that would normally be covalently tethered. However, this was accompanied by a dramatic decrease in product yield (Yan et al., 2009) (Fig. 2e). This may indicate the presence of secondary interactions at this interface between the ACP and KS domains, which are essential for productive interaction.

#### Class 2 cis-AT PKS DDs: Eight α-helix bundles

**Fig. 3.**
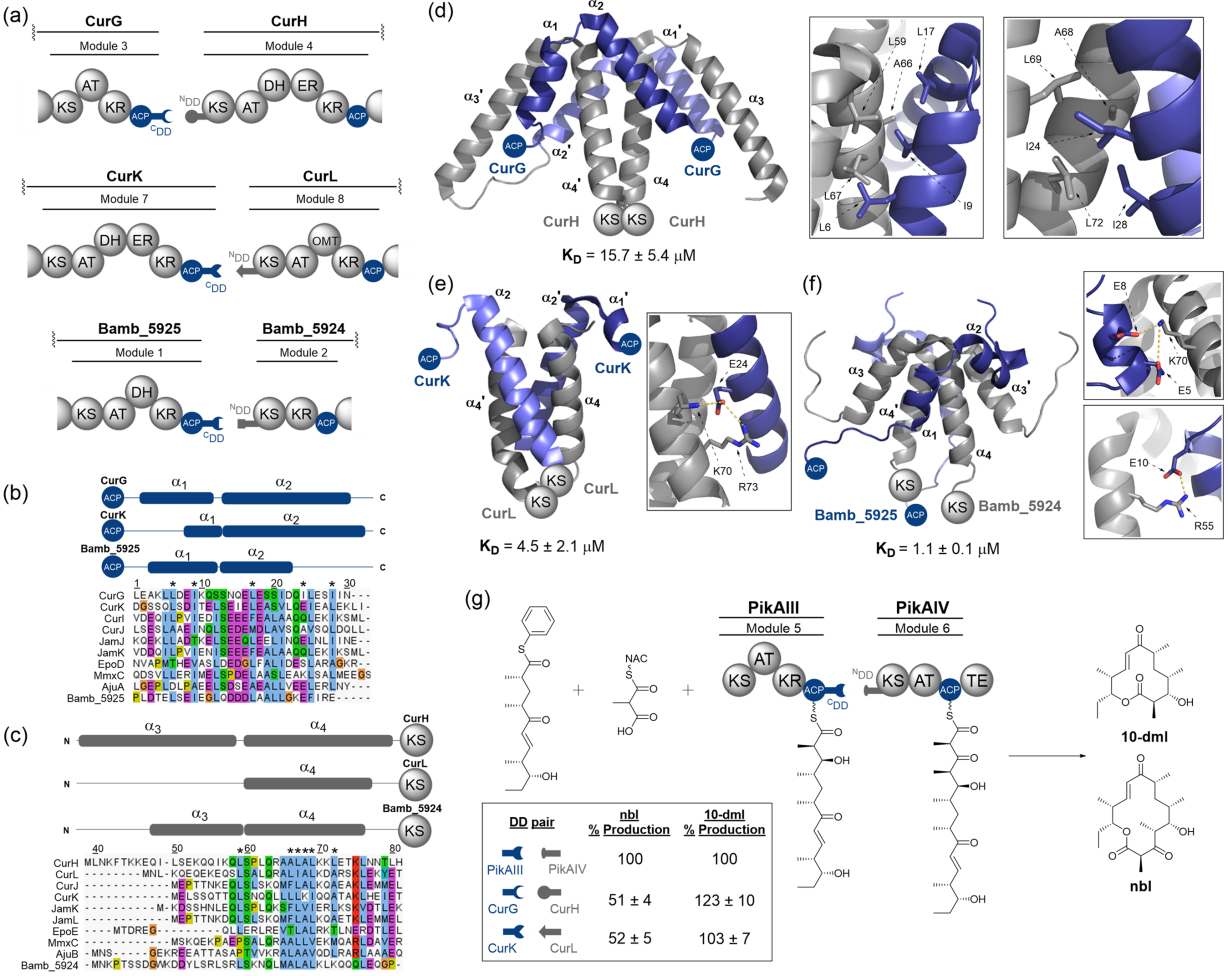
Structural features, sequence alignments and use in biosynthetic engineering of PKS Class 2 DDs. (a) Domain architecture of the CurG-CurH, CurK-CurL and Bamb_5925-Bamb_5924 intersubunit junctions from the hybrid PKS-NRPSs responsible for the biosynthesis of curacin A and enacyloxin IIa. Cognate DD pairs are depicted using complimentary fitting shapes. (b) Sequence alignment of select eight α-helix bundle C-terminal DDs. (c) Sequence alignment of select eight α-helix bundle N-terminal DDs. Asterisks (*) denote the positions of the conserved hydrophobic interfacial residues highlighted in (d). Above the alignments, schematics displaying the positions of the secondary structural elements observed in the structures of each of the DD complexes is provided. (d) X-ray crystal structure of the eight α-helix bundle docked complex formed by the covalently tethered CurG C-terminal and CurH N-terminal DDs (PDB accession code: 4MYY). *Inset:* Hydrophobic residues across α_1_, α_2_, α_4_, α_3_′ and α_4_′ implicated in the formation of the CurG–CurH DD interface. (e) X-ray crystal structure of the docked complex formed by the covalently tethered CurK C-terminal and CurL N-terminal DDs (PDB accession code: 4MYZ) *Inset:* Electrostatic interactions between the α_2_′ and α_4_ helices. The same interactions are not conserved in the CurG–CurH interface. (f) Solution state NMR structure of the docked complex formed by the covalently tethered Bamb_5925 C-terminal and Bamb_5924 N-terminal DDs (PDB accession code: 6TDN) *Inset:* Electrostatic interactions between α_1_ and α_4_ (*top*) and α_1_ and α_3_ (*bottom*). The former is also observed in the CurG–CurH interface. Residue numbering in (b)–(e) is relative to that of PDB entry 4MYY. (g). Engineering of the PikAIII–IV intersubunit junction from the pikromycin PKS. This PKS produces two products, pikromycin, of which narbolide (nbl) is the precursor, and methymycin, for which 10-deoxymethynolide (10-dml) is the precursor, resulting from a module-skipping mechanism. Exchanging the WT four α-helix bundle DD pair at this junction with the eight α-helix bundle CurG-H or CurK-L DD pair was demonstrated to maintain productive interaction between proteins by *in vitro* assays. Additionally, introducing eight α-helix bundle DDs was found to change the product profile from 50% nbl, to predominantly 10-dml, suggesting more effective delivery of the PikAIII ACP-tethered substrate to the PikAIV TE domain. Percentage production values are given with respect to the WT DD pair.

There are three characterised examples of Class 2 PKS DDs: two from the curacin modular PKS at the CurG/H and CurK/L subunit junctions (Whicher et al., 2013), and one from the enacyloxin hybrid PKS–NRPS at the Bamb_5925/5924 subunit junction (Risser et al., 2020). All characterised examples of Class 2 DDs form dimeric eight α-helix bundles, with each DD contributing two α-helices (Fig. 3a–c). Much like Class 1 PKS DDs, a parallel coiled–coil formed by the first ^N^DD α-helix interacting with its symmetry mate (α_4_ and α_4_′) is at the centre of the bundle. The two ^C^DD α-helices both interact with the ^N^DD coiled–coil. This interaction interface is predominantly hydrophobic, with salt bridges hypothesised to confer specificity (Fig. 3d–f). However, the sites of these specific interactions appear to vary. For example, interactions for the CurK/L complex, as highlighted in Fig. 3e, are between E24/K70 and E24/R73 (Whicher et al., 2013). In Bamb_5925/24 K70 forms salt bridges with E5 and E8 on helix α_1_ (Fig. 3f), rather than an amino acid on helix α_2_ as in the CurK/L complex (Risser et al., 2020) (Fig. 3e). Furthermore, a productive electrostatic interaction does not exist at these sites for the CurG/H interface (Fig. 3d). There is a much greater level of conservation at the positions of hydrophobic contact across all characterised examples of Class 2 DDs (Fig. 3b and c). For example, the hydrophobic amino acids highlighted in CurG/H in Fig. 3d; L6, I9, L17, L59, A66 and L67, are conserved in both CurK/L and Bamb_5925/24 (Fig. 3e and f).

There is a high level of structural similarity between CurG/H and Bamb_5925/24. However, CurK/L has a much shorter primary ^C^DD α-helix (α_1_) and lacks the primary ^N^DD α-helix (α_3_) observed for CurH and Bamb_5924 ^N^DDs. Sequence analysis shows that three N-terminal amino acids are missing from CurL, which may prevent the ^N^DD from folding correctly. However, structurally the CurK/L complex appears closer to that of a Class 1 four α-helix bundle than that of a Class 2 system, despite its sequence aligning well to those of other Class 2 PKS DDs (Risser et al., 2020; Whicher et al., 2013). Once the structures of additional DD pairs have been elucidated, it will become clear whether Class 2 PKS DDs should be split into further subcategories according to their docked complex structure.

Despite the overall similarity of Bamb_5925/24 to CurG/H, there are several distinct differences between these two DD pairs (Risser et al., 2020). The second α-helix of the Bamb_5924 ^N^DD adopts a very different orientation on docking compared to the CurH ^N^DD (Risser et al., 2020). Furthermore, all four ^N^DD α-helices interact at the coiled–coil in Bamb_5924, compared to only those involved in coil formation (α_4_ and α_4_′) in CurH (Risser et al., 2020). Finally, Bamb_5924 has hydrophobic residues at sites that are hydrophilic in CurH (Risser et al., 2020). These factors all contribute to a more compact eight helix bundle at the Bamb_5925/24 interface compared to CurG/H (Risser et al., 2020). This may be a feature common to eight α-helix bundles in hybrid PKS–NRPSs.

Interestingly, the CurK ^C^DD was found to be promiscuous, interacting with both CurH and CurM ^N^DDs, albeit with a reduced affinity (Whicher et al., 2013). This observation is difficult to rationalise based on inspection of the amino acid sequences alone. However, it represents the sole example of PKS DDs working outside the confines of their cognate pairs and suggests that productive DD interactions may not be solely responsible for maintenance of biosynthetic fidelity. Substrate specificity and gating, or domain–domain interactions across subunit junctions are also likely to be important in productive protein–protein interactions.

Engineering of the pikromycin system was undertaken using curacin DD pairs; the CurG/H and CurK/L pairs were used to replace native DDs at the PikAIII/IV interface (Fig. 3g) (Whicher et al., 2013). The pikromycin PKS produces two products: 10-deoxymethynolide (10-dml), a methymycin precursor, and narbonolide (nbl), a pikromycin precursor. While overall product yield remains constant on exchange of Class 1 PKS DDs with their Class 2 counterparts, the product profile changes from 50% nbl to predominantly 10-dml (Whicher et al., 2013). This indicates that Class 2 DDs may promote different subunit interactions to Class 1 DDs, perhaps demonstrating that they permit increased sampling of the terminal end of the downstream module.

### *Trans-*AT PKS DDs

Unlike *cis*-AT systems, *trans*-AT PKSs possess both inter- and intramodular subunit junctions. Consequently, these subunit junctions are located between a variety of domains, giving rise to ‘split module’ architectures such as KS/KR, KR/DH and KS/DH domain interfaces, amongst others (Dorival et al., 2016; Jenner et al., 2018; Zeng et al., 2016). There are two key classes of DDs reported in *trans*-AT PKSs which account for a large number of the subunit junctions: the four α-helix bundle DDs and dehydratase docking (DHD) domains.

#### Four α-helix bundles

Four α-helix bundle DDs were identified as regions of ∼25 amino acids at the N- and C-termini of protein subunits in *trans*-AT PKSs. While these DD regions occur at traditional intermodular ACP/KS subunit junctions, they are also found at many of the unusual intramodular subunit junctions responsible for ‘split module’ domain architectures (Dorival et al., 2016; Zeng et al., 2016).

**Fig. 4.**
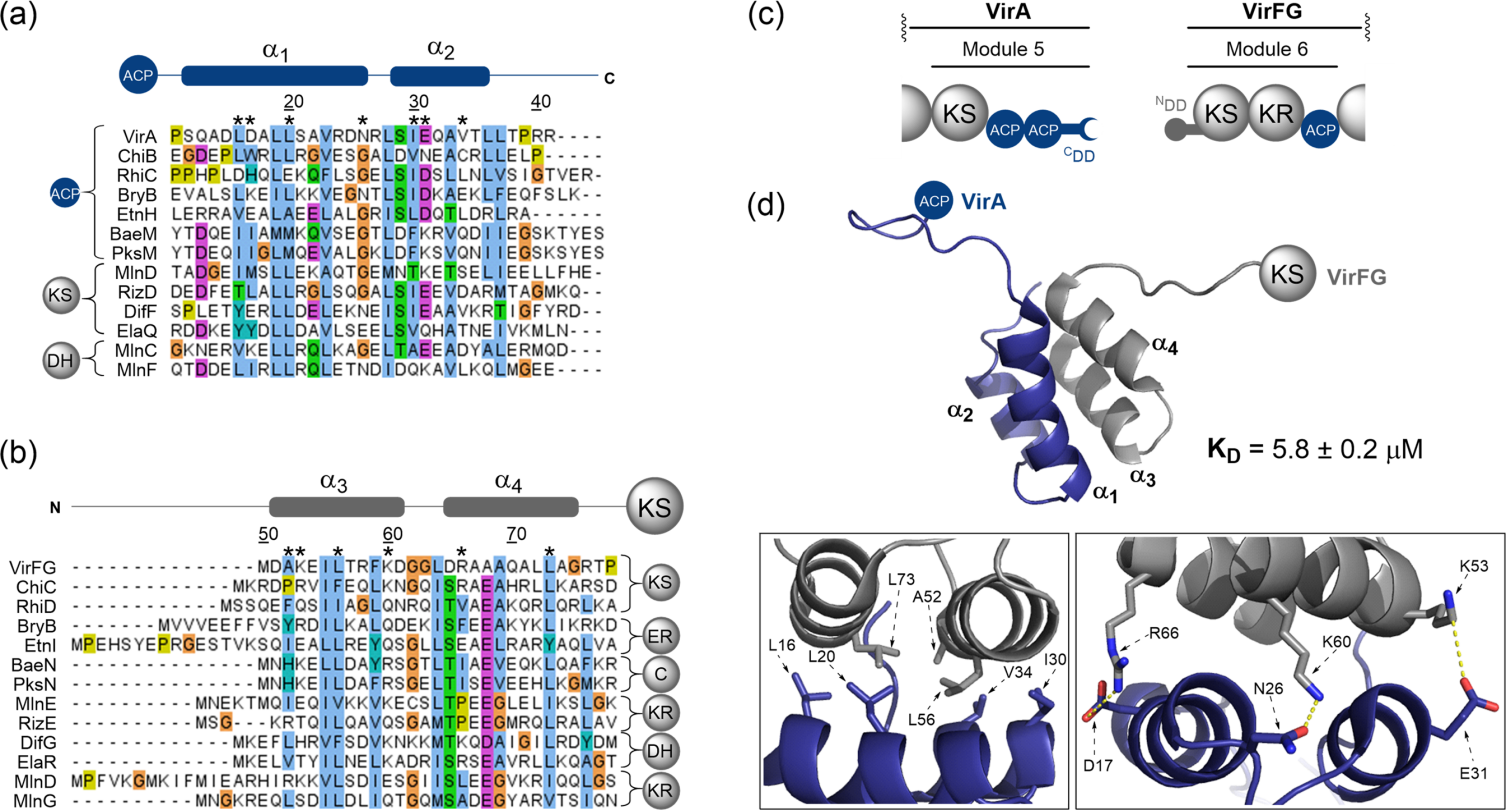
Structural features and sequence alignments of PKS four α-helix bundle DDs. (a) Sequence alignment of select ^C^DDs. (b) Sequence alignment of select ^N^DDs. Residue numbering throughout is relative to that of PDB entry 2N5D. Asterisks (*) denote the positions of the interfacial residues highlighted in (d). Above the alignment, a schematic displaying the positions of the secondary structural elements observed in the VirA–VirFG docked complex is provided. (c) Domain architecture of the VirA–VirFG intersubunit interface in the virginiamycin *trans*-AT PKS. (d) Solution state NMR structure of the docked complex formed by covalently-tethered VirA ^C^DD and VirFG ^N^DD (PDB accession code: 2N5D). *Inset (left)*: Hydrophobic interface formed between all four helices. *Inset (right)*: Electrostatic and hydrogen bonding interactions at the docking domain interface.

The VirA–FG ACP/KS interface in the virginiamycin PKS was the first characterised example of a four-helix bundle DD (Dorival et al., 2016). Solution state NMR spectroscopy of the covalently tethered ^C^DD–^N^DD complex elucidated both the docked structure and molecular interaction mechanisms involved in this interface. In the docked complex, both the ^N^DD and ^C^DD regions adopt two α-helices, forming a helix-turn-helix motif on each side of the four α-helix bundle. Two sets of α-helices are offset by 127° in the complex, producing an interaction interface that encompasses all four α-helices (Fig. 4c and d) (Dorival et al., 2016). While *trans*-AT PKSs are indeed dimeric, SAXS data suggests that this does not result in dimerisation of the ^C^DD and ^N^DD regions. Instead, there are two separate copies of the VirA–FG DD pair. Consequently, we propose that, rather than describing this interface as a further example of a four α-helical bundle, it should be termed a ‘double-helix pair’. This allows it to be categorised separately from Class 1 *cis*-AT PKS DDs, accounting for differences in both structure and interaction interface (Dorival et al., 2016). The core of the VirA–FG interface is hydrophobic with ^C^DD residues L16, L20, I30 and V34 interacting with A53, L57, F60 and L74 of the ^N^DD, as highlighted in Fig. 4d. Key salt bridges thought to confer specificity are D17/R66, N26/K61 and E31/K54.

Further work investigating intersubunit interactions in the macrolactin *trans*-AT PKS yielded an X-ray crystal structure of the N-terminal KR domain from the MlnE subunit with the ^N^DD appended, allowing visualisation of where the DD sits with respect to the KR domain (Zeng et al., 2016). In this instance the upstream subunit, MlnD, has a KS domain at its C-terminus, an example of the KS/KR ‘split module’ architecture. Other ‘split module’ interfaces in the macrolactin system harbouring DD pairs were examined by analytical gel filtration and isothermal titration calorimetry (ITC) for their ability to form complexes. This showed that non-cognate ^C^DD–^N^DD pairs were unable to form stable complexes (Fig. 4a and b). However, DD swapping experiments showed that cognate ^C^DD–^N^DD pairs could be appended to different catalytic domains and still form a functional complex (Zeng et al., 2016). This highlights the portability of these short DD pairs and their potential utility for biosynthetic engineering efforts (Meinke et al., 2019).

#### DHD domains

**Fig. 5.**
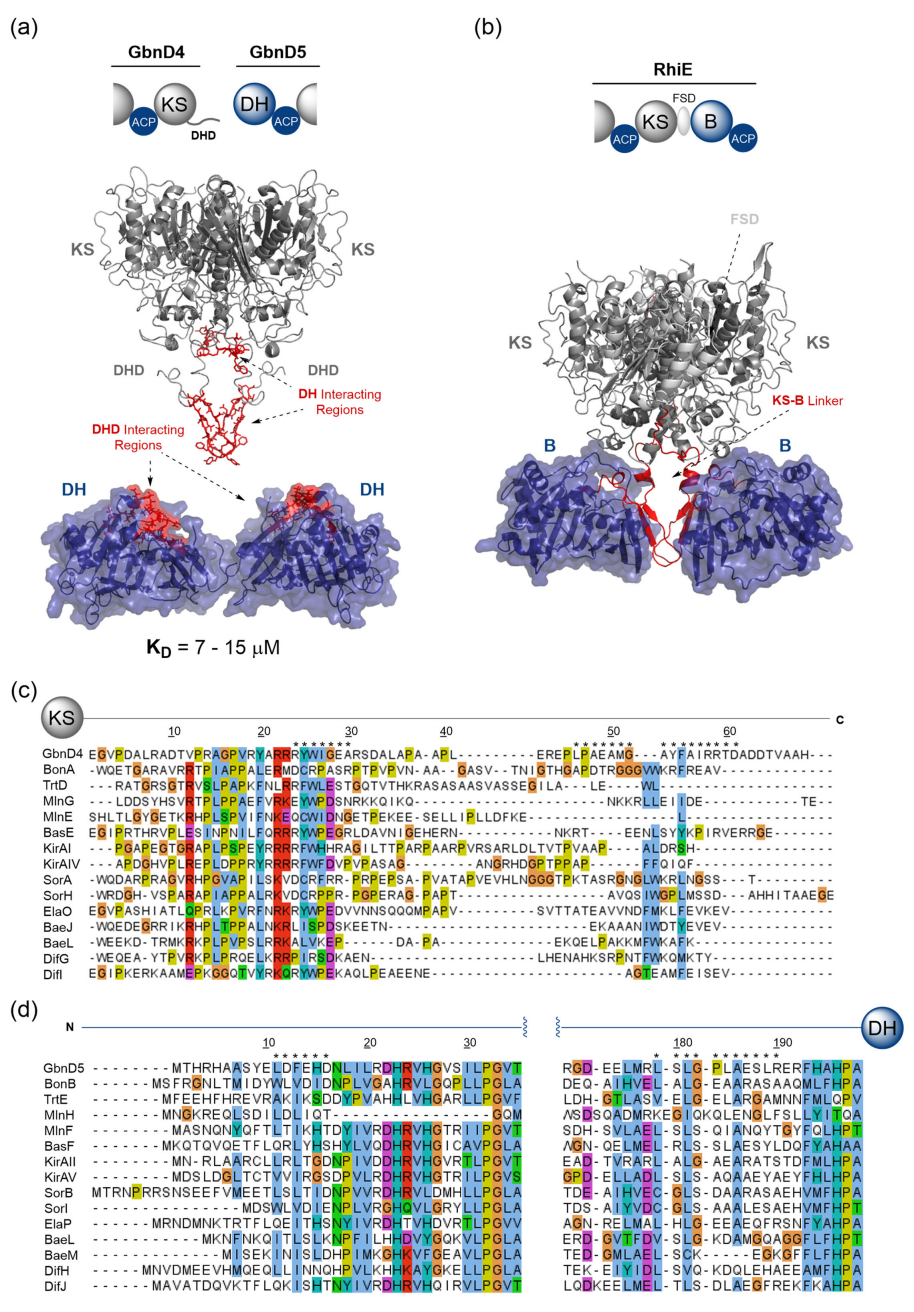
Structural features and sequence alignments of PKS DHD domains and corresponding DH domains. (a) Domain architecture (*top*) and structural model (*bottom*) of GbnD4–GbnD5 KS-DH intersubunit junction from the gladiolin *trans*-AT PKS. Regions highlighted in red on the DHD domain and DH domain have been shown to interact. (b) Domain architecture (*top*) and X-ray crystal structure (*bottom*) of RhiE KS-B di-domain from the rhizoxin *trans*-AT PKS (PDB accession code: 4KC5). The B domain is structurally homologous to a DH domain, and the region connecting the KS to the B domain is highlighted in red. The flanking subdomain (FSD) is highlighted, which is absent from KS domains at KS–DH junctions. (c) Sequence alignment of selected DHD domains from the C-termini of KS domains. (d) Sequence alignment of selected N-terminal DH domains corresponding to the DHD domains in (c). Asterisks (*) denote the positions of the interfacial residues highlighted in (a), as observed experimentally by NMR spectroscopy and carbene footprinting mass spectrometry. Domain abbreviation: B, branching domain.

Recent work on the gladiolin *trans*-AT PKS identified a new class of DD which occurs solely at KS/DH domain junctions (Jenner et al., 2018). Sequence-level inspection of C- and N-termini at these interfaces revealed a ∼70 amino acid region was appended to the C-terminus of the KS domains. However, no additional region could be identified at the N-terminus of the DH domains. *In vitro* acyl transfer and mechanism-based crosslinking experiments showed that the region appended to the C-terminus of the KS domain was critical for functional complex formation and was therefore termed a dehydratase docking (DHD) domain. Application of solution state NMR and circular dichroism spectroscopy to the isolated DHD domain revealed a limited propensity to form secondary structure elements in solution, and inherent intrinsic disorder. NMR titrations of the 15N-labelled DHD domain with the DH domain identified two interacting regions, indicating that the former interacts directly to the exterior surface of the latter. This was confirmed by carbene footprinting mass spectrometry analysis, resulting in masking of regions on the DH domain surface upon incubation with the DHD domain (Jenner et al., 2018) (Fig. 5a, c and d).

Although precise docking orientation and specific amino acid pairwise interactions for this interface are yet to be elucidated, some global insights into the organisation of the KS/DH interface can be inferred from the X-ray crystal structure of the RhiE KS-B di-domain (Bretschneider et al., 2013). The B domain, which in this instance forms a structural scaffold for a chain branching reaction in rhizoxin biosynthesis (Partida-Martinez & Hertweck, 2007), has the same characteristic double-hotdog fold as a DH domain (Keatinge-Clay, 2012). The structure of the di-domain highlights that the KS and the B domains are connected via a long stretch of residues that lack secondary structure elements prior to the start of the B domain, where a set of antiparallel β-sheets form in addition to a short helix (Fig. 5b). Furthermore, the contacts that these parts of the KS-B linker make with the B domain itself correlate well with the interacting regions on the surface of the DH domain identified by carbene footprinting (Fig. 5a).

Interestingly, the KS domain at DHD domain-containing interfaces lacks a flanking subdomain (FSD); a region of protein that is structurally important at the KS–AT interface in *cis*-AT PKSs and is believed to be an evolutionary relic in *trans*-AT PKS systems (Gay et al., 2014, 2016). The significance of this is currently unclear but given that the FSD would naturally precede the DHD domain, it may have been lost to permit close association of the KS and DH domains during complex formation.

## NRPS DDs

NRPSs are responsible for the biosynthesis of non-ribosomal peptide natural products. A minimal NRPS module comprises an adenylation (A) domain, a condensation (C) domain and a PCP domain. A domains select and activate specific amino acids as aminoacyl thioesters. C domains then catalyse amide bond formation between successive PCP-bound aminoacyl thioesters resulting in assembly of a peptide chain. There are two known classes of DD that have been reported to occur exclusively in NRPS systems; communication (COM) domains (Hahn & Stachelhaus, 2004) and peptide-antimicrobial-*Xenorhabdus* (PAX) domains (Watzel et al., 2020). A third class, the β-hairpin docking (βhD) domains have been observed in both NRPSs and hybrid PKS–NRPSs and will be discussed under the hybrid PKS–NRPS section (Dowling et al., 2016; Richter et al., 2008).

### COM Domains: Helix-Hand Motif

**Fig. 6.**
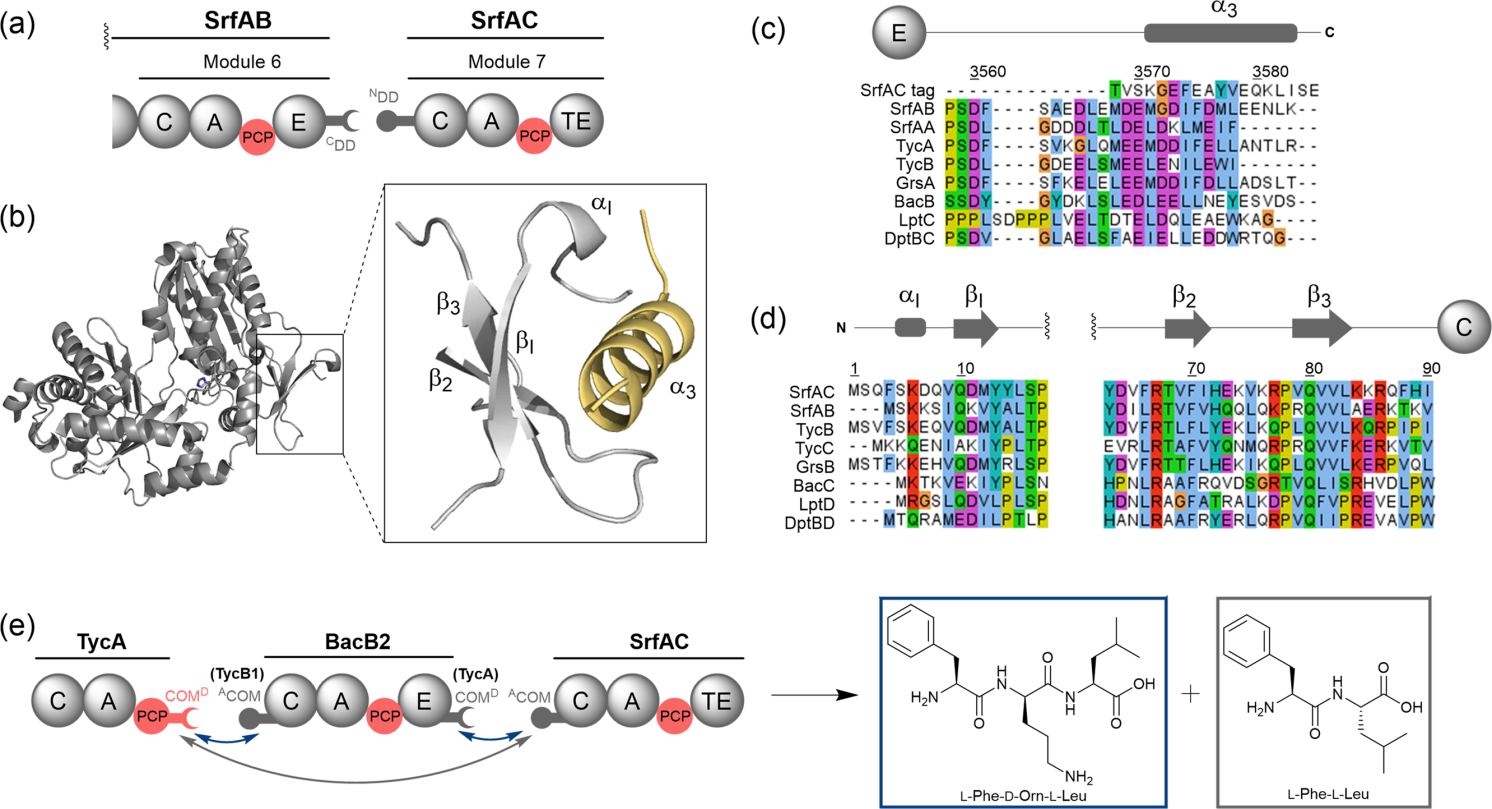
Structural features, sequence alignments and use in biosynthetic engineering of NRPS COM domains. (a) Domain organisation of the SrfAB–SrfAC intersubunit junction. (b) X-ray crystal structure of the SrfAC condensation domain (PDB accession code: 2VSQ). *Inset*: The helix-hand motif is formed of a helix and a beta sheet comprising three non-contiguous strands. A portion of the C-terminal protein tag of SrfAC, shown in yellow, was found to interact with the helix-hand motif and is proposed to mimic the ^N^DD helix. (c) Sequence alignment of selected C-terminal COM^D^ domains. The sequence of the interacting region of the SrfAC C-terminal tag is provided and aligned as described by Tanovic et al. (d) Sequence alignment of selected N-terminal COM^A^ domains, encompassing the two regions of protein comprising the helix-hand motif. Residue numbering throughout is relative to that of PDB entry 2VSQ. (e) Engineering of intermodular interfaces using the TycA–TycB COM domain pairs to mediate productive crosstalk between non-cognate NRPS modules from the tyrocidine, bacitracin and surfactin A assembly lines. Successive interactions of TycAΔE–BacB2 and BacB2–SrfAC indicated by the blue arrows lead to formation of the tripeptide shown in the blue box, while the direct interaction between TycAΔE–SrfAC indicated by the grey arrow leads to the dipeptide product shown in the grey box.

Communication-mediating or COM domains have been identified at multiple E/C domain junctions in NRPSs (Chiocchini et al., 2006; Hahn & Stachelhaus, 2004). As was observed for the DEBS DDs in *cis*-AT PKSs, many experiments were undertaken to increase understanding of the role of COM domains prior to elucidation of their structure (Hahn & Stachelhaus, 2006; Siewers et al., 2010). A possible donor COM (COM^D^) domain structure was obtained relatively serendipitously. Upon crystallisation of the final module of the surfactin NRPS (SrfAC; COM^A^-C-PCP-TE) the structure of the N-terminal acceptor COM (COM^A^) domain was identified (Tanovic et al., 2008). This was somewhat unexpected, as DDs are often highly flexible, unstructured proteins which may only fold in the presence of their partner domain. However, the crystal structure of SrfAC shows the COM^A^ domain entwined with the α-helical myc-His_6_ tag (Fig. 6a and b). On closer inspection the sequence of this tag, used for affinity purification, was found to be similar to that of the upstream SrfAB COM^D^ domain, suggesting this binding mode may be comparable to that of the native complex (Tanovic et al., 2008). Interestingly, the identified COM^A^ region of SrfAC is not just appended to the N-terminus as initially proposed (α_1_, β_1_) (Hahn & Stachelhaus, 2004), it also encompasses two further β-strands (β_3_ and β_4_) embedded within the globular structure of the C domain (Tanovic et al., 2008). These strands form a hand-shaped motif, which acts as a docking site for the COM^D^ domain. This mode of interaction is somewhat similar to that of the DHD domain observed in *trans*-AT PKSs, where a C-terminal donor docking region interacts directly with the surface of the downstream domain (Jenner et al., 2018). Work investigating interaction of the gramicidin S GrsA C-terminal COM domain with tyrocidine TycB N-terminal COM domain via photocrosslinking and subsequent mass spectrometry, provided evidence for a structure similar to that of SrfAC (Dehling et al., 2016). However, the orientation of the COM^D^ domain helix was inverted. This may be an artefact of studying a non-cognate DD pair. Alternatively, this could be a consequence of incorporating a non-native amino acid into the protein, which was necessary for the photocrosslinking.

Engineering experiments using COM domains have exploited their inherent promiscuity. For example, the N-terminal portion of the COM^A^ hand-motif of TycB1 (tyrocidine NRPS) and SrfAC (surfactin NRPS) were found to have 88% sequence identity, while GrsB1 COM^A^ (gramicidin NRPS) had 75% identity. Consequently, all three were found to interact with the COM^D^ α-helix of TycA (tyrocidine NRPS) (Chiocchini et al., 2006). This observation was utilised to generate a triple hybrid NRPS system in *E. coli* (Hahn & Stachelhaus, 2006). Three proteins were used: TycA-COM^D^, COM^A^(TycB)-BacB2-COM^D^(TycA) and SrfAC. As TycA and SrfAC interact, this led to production of dipeptide and tripeptide products (Hahn & Stachelhaus, 2006) (Fig. 6e). However, this work was carried out prior to elucidation of the structure of COM domains. Therefore, only the N-terminal portion of the hand motif (α_1_ and β_1_) was grafted onto BacB2. This innate promiscuity of COM domains is further evidenced by crosstalk experiments employing the surfactin, tyrocidine and gramicidin S NRPS pathways. The non-cognate TycA COM^D^ and TycC COM^A^ domains from the tyrocidine NRPS were used to replace the cognate SrfA-A/SrfA-B COM domain pair in the surfactin NRPS in *B. subtilis* CC112 (Chiocchini et al., 2006). As the TycA COM^D^ domain interacts with the surfactin SrfA-C COM^A^ domain, this led to skipping of SrfA-B and production of a lipotetrapeptide product, rather than the full-length lipoheptapeptide (Chiocchini et al., 2006). Furthermore, *in vitro* work on the gramicidin S NRPS showed GrsA was able to interact directly with TycB1 from the tyrocidine NRPS resulting in production of cyclo-d-Phe-l-Pro-diketopiperazine (Torsten Stachelhaus et al., 1998). The alignments provided in Fig. 6c and d show a high level of sequence conservation across all COM^D^ and COM^A^ domain pairs. Therefore their inherent promiscuity is perhaps unsurprising.

As the COM^A^ domain is not wholly independent of its tethered catalytic domain (i.e. structural elements are contributed from other sections of the polypeptide chain), the question of whether it would be possible to cleanly ‘cleave’ COM^A^ regions for NRPS pathway engineering remains unclear. For engineering purposes, it may be more productive to focus on utilising the inherent promiscuity of COM domains and on COM^D^ domain-based grafting studies, before attempting COM^A^ domain-based engineering.

### PAX DDs: Three α-Helix Bundles

**Fig. 7.**
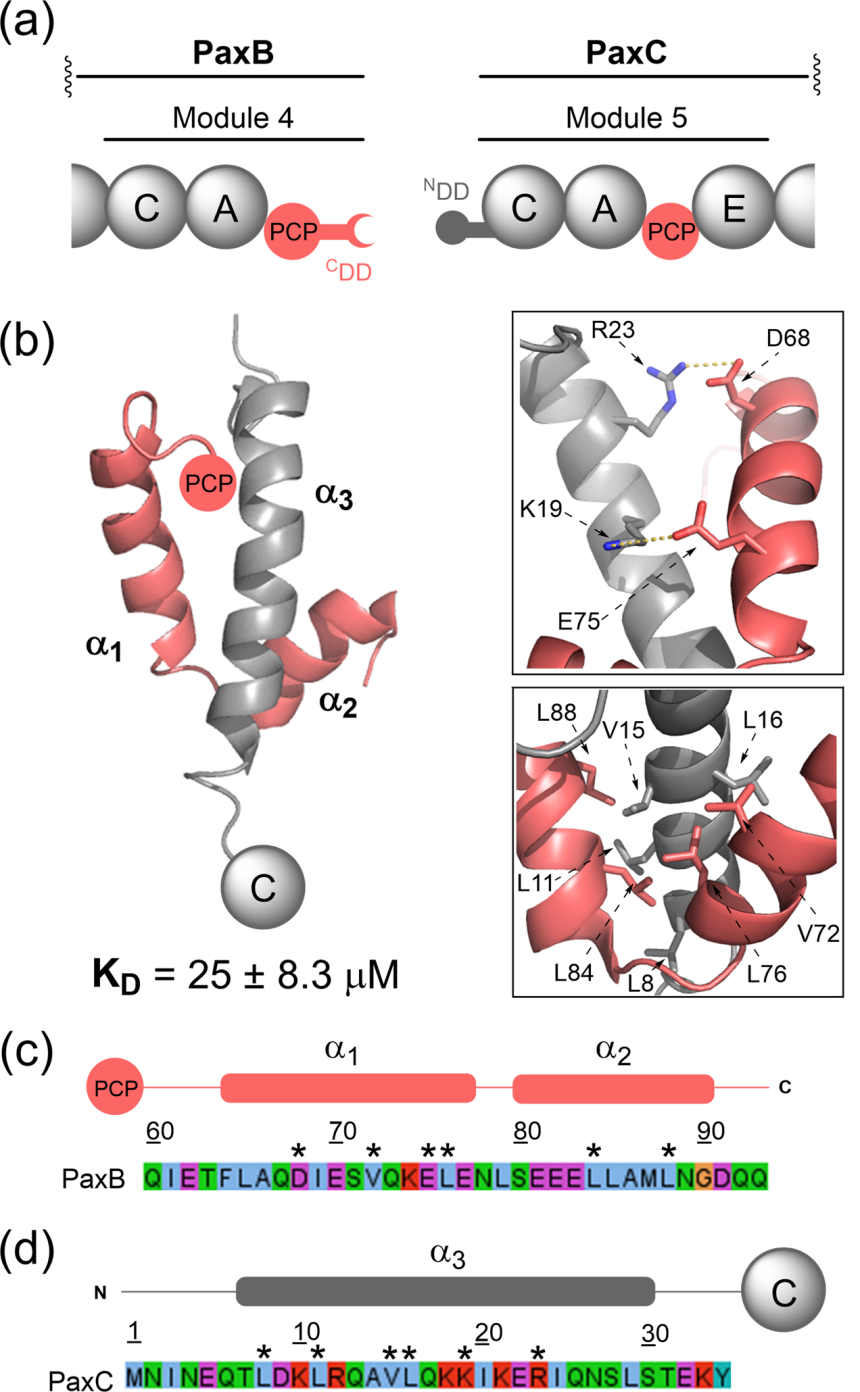
Structural features and sequence alignments of NRPS three α-helix bundle DDs. (a) Domain organisation of the PaxB–PaxC intersubunit junction. (b) Solution state NMR structure of the docked complex formed by the covalently tethered PaxB ^C^DD and PaxC ^N^DD (PDB accession code: 6TRP). *Inset (top):* Electrostatic interactions between the α_1_ and α_3_ helices. *Inset (bottom)*: Hydrophobic interface formed between all three helices. (c) Sequence of the PCP domain-tethered PAX ^C^DD. (d) Sequence of the C domain-tethered PAX ^N^DD. Asterisks (*) denote the positions of the interfacial residues highlighted in (b). Above each sequence, a schematic displaying the positions of the secondary structural elements observed in the docked complex is provided.

A new class of NRPS DD was recently identified via analysis of the PaxB-C PCP/C domain junction from the PAX assembly line (Watzel et al., 2020) (Fig. 7a). Solution state NMR spectroscopy of a covalently tethered ^C^DD–^N^DD complex illuminated both the structure of the DDs and the molecular interactions across the interface. The ^N^DD comprises a single α-helix which docks centrally within the two α-helix ^C^DD to give a three α-helix bundle with a V-shaped overall structure (Fig. 7b) (Watzel et al., 2020). As for most other DDs, salt bridges confer specificity, in this case K19/E75 and R23/D68. The remaining interactions are hydrophobic. Key hydrophobic residues include L8, L11, V15 and L16 on the ^N^DD and L72, L76, L84 and L88 on the ^C^DD (Fig. 7b–d). Bioinformatics analyses predicted further examples of this class of DD bound to C, PCP, ACP and oxidoreductase domains, showing their portability and potential utility for NRPS pathway engineering (Watzel et al., 2020).

## Hybrid PKS–NRPS DDs

Hybrid PKS–NRPSs assemble mixed polyketide/non-ribosomal peptide natural products composed of both amino acids and (alkyl)malonyl-derived building blocks. These systems illustrate that, with the right tools, it may be possible to engineer pathways involving both NRPS and PKS biosynthetic machinery, greatly increasing the diversity of the products.

### β-Hairpin DDs

**Fig. 8.**
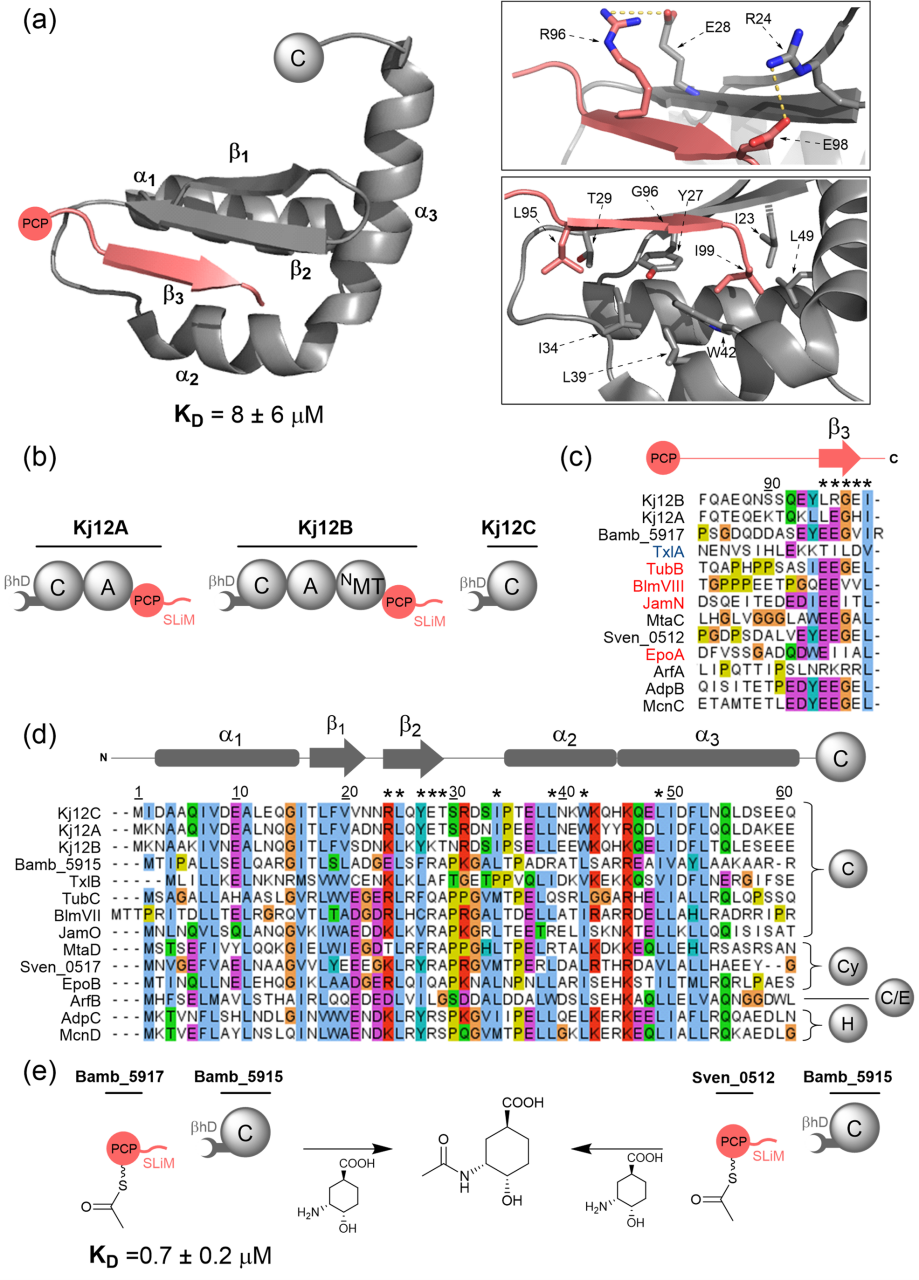
Structural features, sequence alignments and use in biosynthetic engineering of βhD domains. (a) Solution state NMR structure of the docked complex formed by the covalently tethered Kj12B C-terminal SLiM and Kj12C N-terminal βhD domain (PDB accession code: 6EWV). *Inset (top)*: Residues involved in salt bridge-forming interactions between the SLiM (β_3_) and β_2_ strand of the βhD domain. *Inset (bottom)*: Hydrophobic interface formed between β_2_, β_3_, α_2_ and α_3_. (b) Domain architecture of the rhabdopeptide-producing NRPS. Note that this iterative system contains three βhD domains and two SLiMs, which can all interact with varying affinities. (c) Sequence alignment of select ^c^DD SLiMs. Highlighted in red are SLiMs appended to the carrier protein domain of a PKS module, those in black are appended to the carrier protein domain of NRPS modules. TxlA, blue, contains a SLiM downstream of an E domain. (d) Sequence alignment of select N-terminal βhD domains. To the right of the alignment, the domain directly downstream of the βhD domain in each protein is shown. In (c) and (d), a schematic indicating the positions of the secondary structural elements from the solution state NMR structure is provided. Residue numbering in (a)–(d) is relative to that of PDB entry 6EWV. In (c) and (d), asterisks (*) denote the positions of the key interfacial residues highlighted in (a). (e) SLiM–βhD domain junction in the enacyloxin NRPS–PKS pathway. Crosstalk of the Sven_0512 PCP domain, a SLiM-bearing carrier protein domain from the watasemycin NRPS, with the enacyloxin Bamb_5915 βhD-C domain from the enacyloxin PKS-NRPS was able to produce *N*-acetyl (1*S*,3*R*,4*S*)-3-amino-4-hydroxycyclohexane-1-carboxylic acid. Domain abbreviations are as follows: Cy, heterocyclisation domain; C/E, dual condensation–epimerisation domain; H, flavin-dependent halogenase.

β-Hairpin DDs (βhD domains) have been identified in both purely NRPS and hybrid PKS–NRPS pathways, at PCP/C or PCP/Cy domain interfaces (Dowling et al., 2016; Hacker et al., 2018; Kosol et al., 2019; Richter et al., 2008). An intrinsically disordered short linear motif (SLiM) ^C^DD of less than 15 amino acids, interacts with the much larger N-terminal βhD domain (Hacker et al., 2018; Kosol et al., 2019). This class of DD is perhaps the most well characterised with structures of six different βhD domains deposited in the PDB; three from the rhabdopeptide NRPS (solution state NMR structures) (Hacker et al., 2018), one from the tubulysin PKS–NRPS (solution state NMR structure) (Richter et al., 2008), one from the epothilone PKS–NRPS (X-ray crystal structure) (Dowling et al., 2016) and one from the enacyloxin IIa PKS–NRPS (X-ray crystal structure) (Kosol et al., 2019) (Fig. 8a and b). The structure of the N-terminal βhD domain was found to be highly conserved across all systems adopting an αββαα fold with a central β-hairpin.

βhD domains are connected to the downstream domain via flexible linker regions of approximately 20 amino acids (Dowling et al., 2016; Kosol et al., 2019). The EpoB crystal structure shows the βhD domain in three distinct conformations relative to the downstream Cy domain, with no contacts observed between the DD and the catalytic domain (Dowling et al., 2016). The Bamb_5915 crystal structure shows a further unique conformation of the βhD domain relative to its downstream domain. This suggests βhD domains are inherently mobile with respect to the tethered catalytic domain. However, the role of this mobility remains unclear. Sampling of multiple conformations may aid recruitment of a SLiM binding partner, with the flexible linker then allowing delivery of the carrier protein-bound substrate to the active site of the catalytic domain (Kosol et al., 2019).

Studies of the rhabdopeptide NRPS defined the first structure of a SLiM–βhD domain complex. Solution state NMR spectroscopy of a covalently tethered Kj12B-C ^C^DD–^N^DD complex found that only the final five amino acids of the SLiM interact with the βhD domain (Hacker et al., 2018). These amino acids form a short β-strand lying antiparallel to, and interacting with, the β_2_ strand of the βhD domain via two key salt bridges; E28/R96 and R24/E98 (Hacker et al., 2018) (Fig. 8a). Mutation of the charged β_2_ residues to alanine disrupted binding of the DDs, consistent with similar studies on the tubulysin SLiM–βhD domain interface (Richter et al., 2008). Charged residues at these positions are ubiquitous across such interfaces (Fig. 8c and d). The opposing face of the SLiM β-strand forms a conserved hydrophobic interface with α_2_, α_3_ and β_2_ of the βhD domain (Fig. 8a and d), as confirmed by solution state NMR titrations (Kosol et al., 2019; Richter et al., 2008).

SLiM DDs have been observed to show a level of promiscuity; one SLiM peptide is able to recruit multiple downstream domains with βhD domains appended. For example, the rhabdopeptide NRPS shown in Fig. 8b contains two SLiMs and three βhD domains (Hacker et al., 2018). Each SLiM–βhD domain pair can interact productively (with the exception of Kj12A SLiM and Kj12A βhD domain), allowing iterative functioning of the NRPS modules, leading to production of multiple products. SLiM–βhD domain interfaces have also been demonstrated to undergo crosstalk *in vitro*. The Sven_0512 PCP domain from the watasemycin NRPS, which has a SLiM appended to its C-terminus, has been demonstrated to interact productively with the Bamb_5915 C domain via its βhD domain (Fig. 8e). This is despite the fact that the native partner βhD domain from the watasemycin system has only 35% identity to that of the corresponding domain from Bamb_5915. Addition of excess Sven_0512 SLiM peptide inhibits turnover, demonstrating the SLiM–βhD domain interaction plays an important role in product formation (Kosol et al., 2019). The inherent promiscuity of SLiM–βhD domain interfaces has the potential to be exploited for biosynthetic engineering. However, direct interactions between the carrier protein and the catalytic domain must also be considered to build efficient hybrid pathways.

Several engineering experiments involving βhD domains and associated interfaces have already been undertaken. Engineering of the rhabdopeptide producing NRPS to alter its product profile involved replacement of Kj12A/B and Kj12B/C with *Xenorhabdus*-derived SLiM–βhD domain pairs (XabA/B and XabB/C) from xenoamicin producing pathways in an attempt to prevent module iteration (Cai et al., 2019). Despite replacement with cognate DD pairs, each successive modification was found to decrease the product yield (Cai et al., 2019). This may indicate the importance of maintaining the overall charge of the ^C^DD SLiM in engineering experiments.

**Fig. 9.**
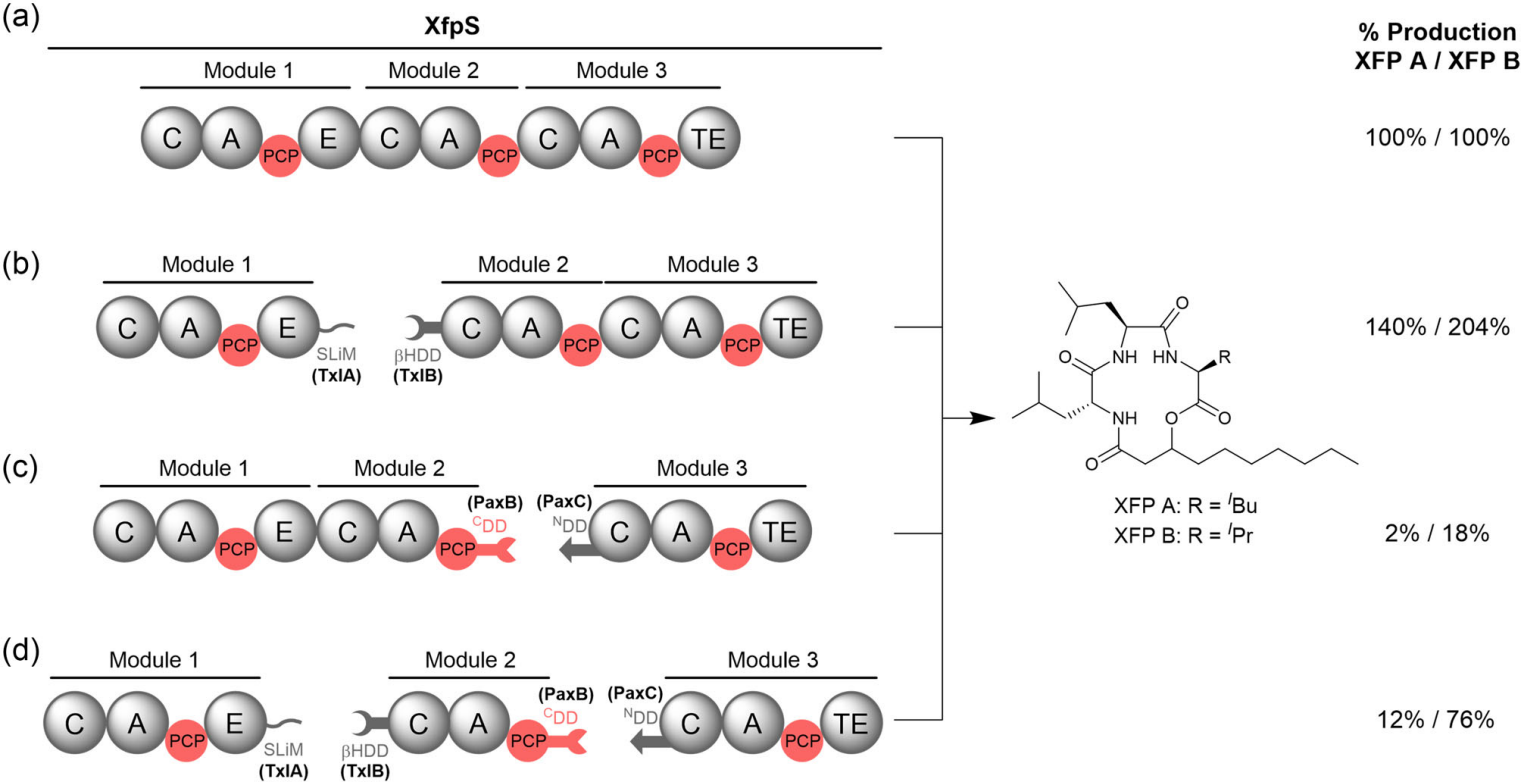
Engineering of XfpS, the single-subunit NRPS responsible for production of xefoampeptides A and B. (a) Wild-type XfpS. *Bottom:* XfpS engineered with DD pairs to artificially split the three modules into separate subunits. (b) As in (a) with a SLiM–βHD domain pair from the TxlA–TxlB intersubunit junction from taxlllaid biosynthesis introduced between modules 1 and 2. (c) Same as (a) with a 3-α-helix bundle DD pair from the PaxB–PaxC intersubunit junction from PAX peptide biosynthesis introduced between modules 2 and 3. (d) Same as (a) with both intermodular junctions engineered as in (b) and (c). In each case, the proteins were heterologously expressed in *E. coli* and XFP A/XFP B production was determined from LC–MS of the methanolic extract. Percentage production is given with respect to the wild-type system in (a). In all cases, a Strep-Tag II affinity tag is found at the N-terminus of module 1 and the C-terminus of module 3.

More recently DDs have been used to split the single trimodular xefoampeptide NRPS protein subunit into three separate proteins (Kegler & Bode, 2020) (Fig. 9). Two junctions were engineered: an E/C domain junction, where the SLiM–βhD domain pair from the TxlA/B interface of the taxlllaid NRPS was grafted, and a PCP/C domain junction where the PaxB/C DDs were tethered. Insertion of PAX DDs greatly decreased product titres, but use of the SLiM–βhD domain pair was found to increase product yield (Kegler & Bode, 2020). It has been acknowledged that protein solubility could contribute to these results. Regardless of whether this is the case, the use of a βhD domain at this cut site to engineer NRPS systems appears promising.

Work on the enacyloxin βhD domain led to identification of more than 1400 examples of SLiM–βhD domain pairs across various domain interfaces (Kosol et al., 2019). SLiMs were identified at the C-terminus of oxidases, heterocyclisation domains, PCP and ACP domains, while βhD domains were identified at the N-terminus of C, E, Cy, TE and MT domains, thioester reductases and halogenases, indicating the prevalence of this type of docking motif. Furthermore, additional examples of one SLiM peptide recruiting more than one downstream subunit to the same PCP domain, as in the rhabdopeptide NRPS, were identified (Kosol et al., 2019). This indicates the potential of SLiM–βhD domain pairs to be employed in genetic engineering of multiple types of system to produce analogues of polyketides, non-ribosomal peptides or hybrids thereof.

## Other Docking Tools

Work in other areas of biochemistry and biotechnology has led to the development of novel protein docking tools that show potential for engineering of natural product pathways. Synthetic biology databases are beginning to be developed, which provide well-characterised molecular components that have the potential to be utilised to further engineer biosynthetic pathways alongside DDs.

### SYNZIPs

SYNZIPs are a synthetic biological tool developed via computational methods that enable engineering of protein–protein interactions (Grigoryan et al., 2009; Park et al., 2017; Thompson et al., 2012). The original development of this tool was inspired by basic-region leucine zippers (bZIPs); transcription factors with a high level of basic amino acids that allow binding of DNA (Grigoryan et al., 2009). There are around 53 bZIP proteins in humans. These are implicated in many biological processes and are therefore attractive targets for inhibition (Grigoryan et al., 2009). Work to develop protein inhibitors of bZIPs led to production of a set of synthetic bZIPs, or SYNZIPs; coiled–coil peptides that bind favourably to bZIPs, inhibiting their interaction with DNA (Grigoryan et al., 2009; Thompson et al., 2012). The coiled–coil SYNZIPs have a repeating seven residue, or heptad pattern (*abcdefg*), containing hydrophobic amino acids, usually at positions *a* and *d*, or *e* and *g* (Park et al., 2017; Thompson et al., 2012). On assessing the developed library of SYNZIPs for pairwise interactions amongst themselves, certain pairs were shown to bind with high affinity, forming coiled–coil interaction interfaces (Thompson et al., 2012).

**Fig. 10.**
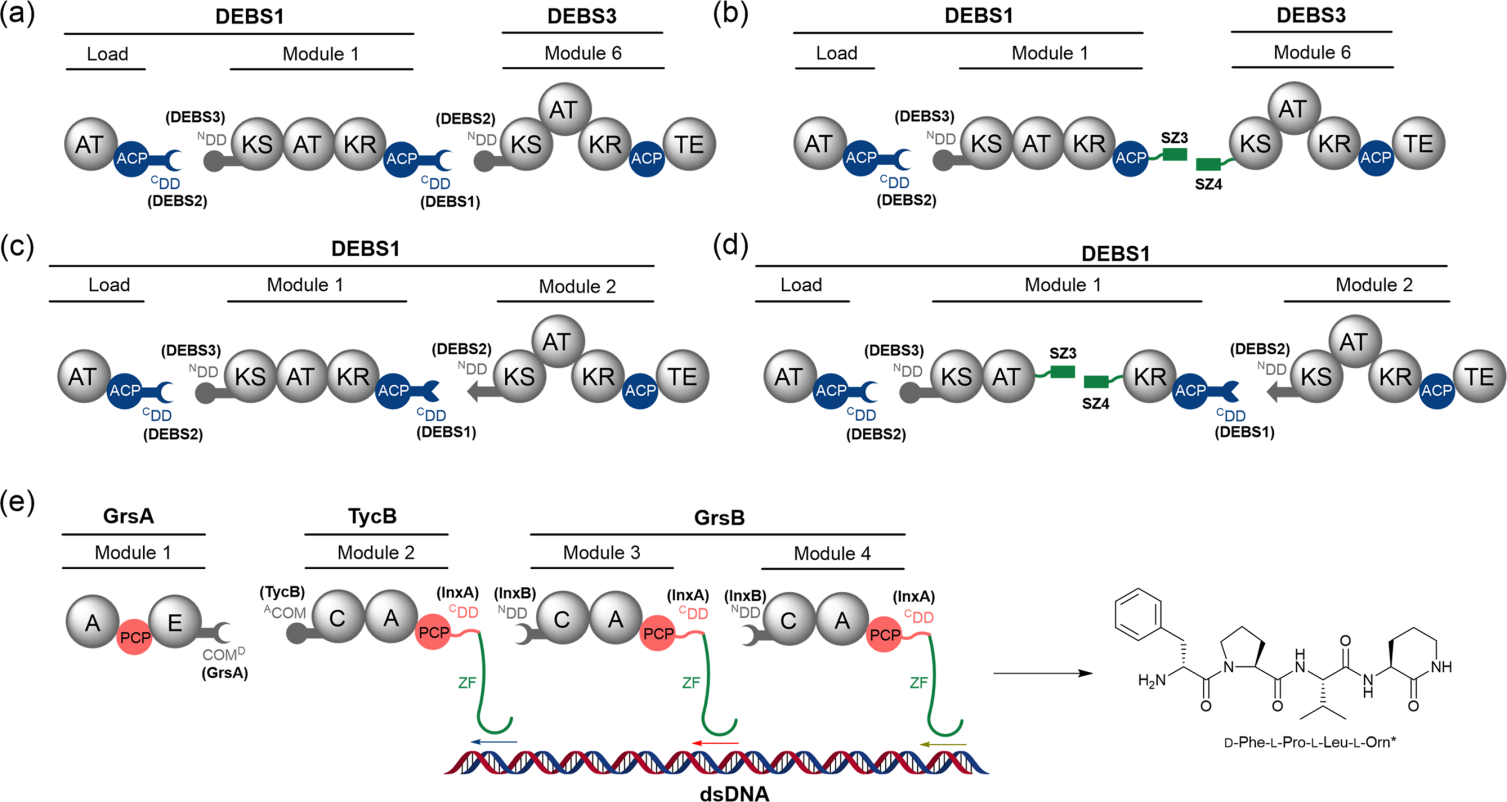
Engineering of PKS and NRPS biosynthetic systems using synthetic docking tools. (a) DEBS1 loading module, module 1 and DEBS3 module 6 engineered using four α-helix bundle DD pairs from DEBS2-3 and DEBS1-2 intersubunit junctions. (b) Same as (a) but with the four α-helix bundle DDs at the module 1-module 6 junction replaced by synthetic DD pair SYNZIP3-4. Higher initial rates of turnover are observed compared to (a). (c) DEBS1 loading module, module 1 and module 2 engineered using four α-helix bundle DD pairs from DEBS2-3 and DEBS1-2 intersubunit junctions. (d) Same as (c) but with module 1 artificially split at the AT–KR junction using the SYNZIP3-4 pair. Initial rates of turnover observed are comparable to (c). In (a–d), the TE domain is derived from the DEBS system and fused to the end of the terminal module. (e) Engineering of the gramicidin S NRPS using DNA templating to direct protein–protein interactions. SLiM–βHD domain pairs from the InxA–InxB intersubunit junction of the rhabdopeptide/xenortide- like peptide biosynthetic pathway from *Xenorhabdus inexxi* were inserted at TycB1–GrsB3 and GrsB3-4 interfaces. ZFs were inserted C-terminal to the SLiMs and allowed site-specific binding to synthetic DNA. The productivity of the engineered system was determined by monitoring production of a known tetrapeptide shunt metabolite that is terminated with a cyclic ornithine residue (denoted as l-Orn*).

Use of high affinity SYNZIP pairs to mediate interaction between PKS modules has been demonstrated. The DEBS PKS has been used as a platform to explore this (Klaus et al., 2019). SYNZIP pairs were able to promote interaction between non-cognate DEBS module 1 and module 6, enhancing turnover compared to use of DDs at this interface (Fig. 10a and b). Furthermore, SYNZIPs enabled artificial splitting of DEBS module 1 between the AT and KR domains achieving turnover similar to that of covalently tethered module 1 (Fig. 10c and d) (Klaus et al., 2019). This shows the potential of SYNZIPs, and other computationally developed protein–protein interaction tools, to advance genetic engineering of multienzymes. With continued optimisation, SYNZIPs may prove to be a valuable tool for biosynthetic engineering.

### DNA-Templating

A recently developed method of engineering protein–protein interactions in natural product pathways involves use of DNA-templating. It is worth noting that DNA-templating has been applied to chemical synthesis for several years, using the principle that single-stranded DNA (ssDNA) templates are tethered to building blocks which, as complementary ssDNA-tags anneal, allows control of the order of connectivity (Goodnow et al., 2017).

Recently, the DNA-templating methodology has been applied to the gramicidin S NRPS (Huang et al., 2020). Here, the multimodular GrsB was split into standalone modules, allowing controlled production of targeted shunt metabolites. A zinc finger (ZF) was tethered to each module. This recognises a specific 9 base pair DNA motif, providing affinity for a double-stranded DNA template which functions as a reaction surface to which the ZF-bearing NRPS modules are bound. A four-module system with three ZF-bound NRPS subunits was generated. A modified SLiM–βhD domain pair from the InxA/B interface from the *Xenorhabdus inexxi* rhapdopeptide/xenortide-like pathway enabled interaction between the ZF-bearing modules, while a non-cognate but interacting COM domain pair enabled interaction between the ZF-lacking GrsA and downstream TycB1 (Fig. 10e). This system was able to achieve turnover at one-third of the rate of wild-type GrsA-B (Huang et al., 2020). However, optimisation of this technique, as already demonstrated via modification of spacing between ZFs on the DNA-template, has the potential to further increase product titre. Replacement of GrsB1 with TycB1, a functionally analogous but more highly expressed protein, may have contributed to pathway inhibition. DNA-templating of NP pathways, while in its infancy, is highly promising, especially as initial data shows it may be possible for it to be used in conjunction with chimeric systems to enhance turnover.

## Conclusions and Future Perspectives

Since identification of the first PKS DD in 1996, termed a linker region rather than a DD at the time, there has been a dramatic increase in the number and type of DDs identified (Aparicio et al., 1996). Whilst some classes of DD have currently only been identified in a single type of multienzyme, bioinformatics analyses have begun to show that certain DD classes are prevalent across PKS, NRPS and hybrid PKS–NRPS systems. There are clearly several different types of DD. Some exist as ^C^DD–^N^DD pairs, such as four α-helix bundles, eight α-helix bundles, double helix pairs and PAX DDs. In others, the ^C^DD interacts directly with the downstream domain, such as helix-hand DDs and DHD domains, or a short peptide ^C^DD interacts with a much larger, structured ^N^DD, as observed in SLiM–βhD domain pairs. Perhaps additional mechanisms of interaction will begin to emerge as protein subunit junctions are studied further.

Experiments using DDs to engineer biosynthetic pathways have increased in success as knowledge of their compatibility and mechanisms of interaction has advanced. How malleable these systems are towards engineering, and the extent to which DDs can help to facilitate this, is not yet fully understood. In most cases, it is still not known what secondary points of contact occur across these junctions, both between pairs of catalytic domains and between DDs and catalytic domains. Once understood, those regions that should be kept consistent to enable conformational changes necessary for protein function will be clarified. This will allow higher product yields to be achieved. For simply bringing two proteins into proximity so as to enable substrate transfer, SYNZIPs are a valuable tool. However, the extent to which synthetic biology tools can be used in parallel to DDs to engineer chimeric systems is only just beginning to be understood.

Another factor to consider is subunit dimerisation, both in the context of NRPSs versus PKSs, but also in *cis-* versus *trans*-AT PKSs. The DDs in each type of system interact differently and this must be considered when using them for engineering. Substrate scope is also an important factor to consider in parallel to DD choice. It transpires that subtle changes are needed in the domains themselves to broaden their substrate scope and enable biosynthetic pathway engineering in its fullest sense. Indeed, recent mutagenesis studies on the active sites of KS domains from the DEBS *cis*-AT PKS to broaden their substrate specificity, showed that KS substrate scope is a crucial factor to consider alongside maintenance of protein–protein interactions to achieve high turnover when undertaking PKS engineering (Klaus et al., 2020)—a phenomenon which has also been observed for KS domains from *trans*-AT PKSs (Jenner et al., 2013, 2015; Nguyen et al., 2008).

In conclusion, understanding of DDs, their structures, key amino acid interactions and the interfaces at which they occur has greatly increased over recent years. Use of DDs to engineer biosynthetic pathways has been undertaken with varying degrees of success. However, factors that must be maintained to successfully engineer a pathway are just beginning to be clearly defined.
